# Genetic Characterization and Diversity of *Rhizobium* Isolated From Root Nodules of Mid-Altitude Climbing Bean (*Phaseolus vulgaris* L.) Varieties

**DOI:** 10.3389/fmicb.2018.00968

**Published:** 2018-05-15

**Authors:** Gilbert Koskey, Simon W. Mburu, Jacinta M. Kimiti, Omwoyo Ombori, John M. Maingi, Ezekiel M. Njeru

**Affiliations:** ^1^Department of Microbiology, Kenyatta University, Nairobi, Kenya; ^2^Department of Forestry and Land Resources Management, South Eastern Kenya University, Kitui, Kenya; ^3^Department of Plant Sciences, Kenyatta University, Nairobi, Kenya

**Keywords:** *Rhizobium*, climbing beans, ARDRA, genetic diversity, 16S rRNA genes

## Abstract

The increasing interest in the use of rhizobia as biofertilizers in smallholder agricultural farming systems of the Sub-Saharan Africa has prompted the identification of a large number of tropical rhizobia strains and led to studies on their diversity. Inoculants containing diverse strains of rhizobia have been developed for use as biofertilizers to promote soil fertility and symbiotic nitrogen fixation in legumes. In spite of this success, there is paucity of data on rhizobia diversity and genetic variation associated with the newly released and improved mid-altitude climbing (MAC) bean lines (*Phaseolus vulgaris* L.). In this study, 41 rhizobia isolates were obtained from the root nodules of MAC 13 and MAC 64 climbing beans grown in upper and lower midland agro-ecological zones of Eastern Kenya. Eastern Kenya was chosen because of its high production potential of diverse common bean cultivars. The rhizobia isolates were characterized phenotypically on the basis of colony morphology, growth and biochemical features. Rhizobia diversity from the different regions of Eastern Kenya was determined based on the amplified ribosomal DNA restriction analysis (ARDRA) of PCR amplified 16S rRNA genes using *Msp* I, *EcoR* I, and *Hae* III restriction enzymes. Notably, native rhizobia isolates were morphologically diverse and grouped into nine different morphotypes. Correspondingly, the analysis of molecular variance based on restriction digestion of 16S rRNA genes showed that the largest proportion of significant (*p* < 0.05) genetic variation was distributed within the rhizobia population (97.5%) than among rhizobia populations (1.5%) in the four agro-ecological zones. The high degree of morphological and genotypic diversity of rhizobia within Eastern Kenya shows that the region harbors novel rhizobia strains worth exploiting to obtain strains efficient in biological nitrogen fixation with *P. vulgaris* L. Genetic sequence analysis of the isolates and testing for their symbiotic properties should be carried out to ascertain their identity and functionality in diverse environments.

## Introduction

Rhizobia bacteria play a significant role in provision of agricultural ecosystem services due to their ability to form symbiotic association with a wide range of leguminous plants that results in biological nitrogen fixation (Orrell and Bennett, 2013). Some of the rhizobia strains are reported to enhance the production of phytohormones, mineral uptake and reduce toxic effects of metals, thereby, indirectly promoting plant growth and development (Karthik et al., 2017) in polluted agricultural soils. Modern agriculture has shifted to the use of sustainable farming practices that are eco-friendly, efficient, and affordable to the resource-limited smallholder farmers. For instance, the use of rhizobia biofertilizers in tropical areas of the Sub-Saharan Africa (SSA) has relatively increased compared to the previous decades due to the agronomic benefits associated with biofertilizers such as yield increase, cost saving, and improved soil health (Meng et al., 2015). However, lack of awareness, absence of supportive infrastructure and limited research targeting the diverse and elite rhizobia strains associated with the newly improved bean lines such as MAC constraints the utilization of biofertilizers in bean production (Ramaekers et al., 2013). In Kenya, limited information on the diversity of rhizobia species that nodulate with the newly released lines of MAC bean varieties and their genetic variation in different AEZ with contrasting environmental conditions is available, hence the need to carry out this study. The data on distribution and genetic variation among the native rhizobia isolates would aid in selecting novel rhizobia strains that could be developed and used as biofertilizers in bean production.

Rhizobia-legume symbiosis is a host-specific association and hence the need to determine the strains and the diversity of rhizobia associated with specific type of legume for better exploitation of the benefits associated with the rhizobia biofertilizers (Batista et al., 2015). Rhizobia distribution and diversity is also affected greatly by the geographical locality and determining their phylogeny could highlight their evolutionary origin. Taxonomically, the diverse heterogenous groups of rhizobia comprise of the alpha group which forms the majority of the rhizobia species and the beta group, which interacts with *Mimosa* genus (Saikia and Jain, 2007). Until recently, about 40 rhizobia species belonging to the seven genera of Alpha-proteobacteria have been identified and includes: *Rhizobium*, *Sinorhizobium*, *Azorhizobium*, *Bradyrhizobium*, *Allorhizobium*, *Mesorhizobium*, *Bradyrhizobium*, and *Methylobacterium* (Lemaire et al., 2015). However, there are other nitrogen fixing bacteria, which have been recently identified from beta and gamma Proteobacteria, that form symbiotic relationship with legumes. Rhizobia from each genera are known to nodulate and fix nitrogen with specific legumes. For instance, *Rhizobium* strains are largely associated with common beans (*Phaseolus vulgaris* L.) and chickpeas (*Cicer arietinum* L.), while *Bradyrhizobium* strains are often found to nodulate soybeans (*Glycine max* L. Merrill), cowpeas (*Vigna unguiculata* L.) and green grams (*Vigna radiata* L.). However, in some cases, cross inoculation by both genera has been documented in some legumes such as mungbeans (*V. radiata* L.) and this is considered advantageous and economical to farmers growing different types of legumes (Hameed et al., 2004).

Like other common beans (*P. vulgaris* L.), the MAC beans establish a mutual and a beneficial symbiotic partnership with *Rhizobium* leading to the formation of root nodules that catalyze nitrogen fixation from the atmospheric air (Baginsky et al., 2015). The species are commonly the fast growing *Rhizobium* and includes; *Rhizobium tropici*, *Rhizobium etli*, *Rhizobium phaseoli*, *Rhizobium leguminosarum*, *Rhizobium gallicum* and *Sinorhizobium meliloti* (Adhikari et al., 2013). The MAC beans were produced through vigorous breeding and were designed to have superior symbiotic nitrogen fixation potential and grain yield output compared to the commonly grown bush beans (Ramaekers et al., 2013). In the recent past, studies investigating the distribution of rhizobia nodulating different legumes in Kenya and other tropical areas of the SSA have revealed a significant diversity in terms of both phenotypic and genotypic traits (Zahran, 2001). This has led to the identification of several distinct groups and the description of novel strains (Berrada et al., 2012; Boakye et al., 2016; Onyango et al., 2015). From these previous researches, there exists a large heterogeneity among the rhizobia strains within the Sub-Saharan region and thus more studies should be channeled on rhizobia distribution, diversity determination and their nitrogen fixation performance.

The tropical soil of the SSA has a great diversity of rhizobia in spite of the pressure on the agricultural resources and harsh climatic conditions which adversely affect the soil ecosystem and its biodiversity (Grönemeyer et al., 2014). In this case, efficient methods of rhizobia classification should be used to fully characterize different rhizobia genotypes and exploit the diverse taxonomic groups that exist in the African tropical areas. Molecular techniques that are more accurate, faster and efficient have been developed to aid the traditional phenotypic and morpho-cultural techniques in distinguishing the different microbial genera, species and strains (Ismail et al., 2013). The polymerase chain reaction (PCR), electrophoresis and gene sequencing tools have revolutionized the characterization of microorganisms by distinguishing even close members of a species up to the strain level based on distinct genetic trait patterns (Baginsky et al., 2015). Moreover, PCR, metagenomics and third generation gene sequencing methods have higher reliability, faster and more sensitive as compared to other conventional methods (Baratto et al., 2012). In other studies, housekeeping genes such as *rec*A and *atp*D, nodulation and symbiotic genes such as *nod*A and *nif*H have been recently used to assess rhizobia diversity and phylogeny in different geographic locations due to their high resolution ability (Ampomah and Huss-Danell, 2016; Wang et al., 2016). This study utilizes PCR and amplified ribosomal DNA restriction analysis (ARDRA) tools to determine the genetic diversity of native rhizobia found in different AEZ of Eastern Kenya. ARDRA involves amplification and digestion of 16S rDNA region using specific restriction enzymes. This method has a higher taxonomic resolution, less expensive and does not require additional equipment used by other fingerprinting techniques such as 16S rRNA gene sequencing (Park et al., 2014).

Apart from ARDRA tools, other PCR-based techniques such as random amplified polymorphic DNA (RAPD), restriction fragment length polymorphism (RFLP) and amplified fragment length polymorphism (AFLP) have been used to evaluate the diversity of rhizobia (Silva et al., 2012; Onyango et al., 2015; Boakye et al., 2016). In addition, the 16S rRNA and 16S-23S rRNA genes, which are highly conserved, have been sequenced to determine the taxonomic position of different rhizobia strains (Rahmani et al., 2011; Adhikari et al., 2013; Karthik et al., 2017). Several rhizobia specific and universal primers have been used in amplification and sequencing and have produced distinguishable rhizobia phylogenies. In recent times, 16S-23S rRNA ITS region is getting consideration among researchers as phylogenetic marker for species and sub-species delineation among rhizobia (Grönemeyer et al., 2014). The high sequence variation of the ITS region allows discrimination between closely related species. According to the PCR-RFLP analysis of 16S-23S ITS region of common bean nodulating rhizobia isolated by Rahmani et al. (2011) in Iran, it was reported that the rhizobia isolated in the study exhibited high genetic diversity and contained 43 ITS genotypes that were clustered into 10 groups at the similarity of 64%.

In this study, we hypothesized that there exists a large heterogeneity among the rhizobia isolates nodulating MAC beans grown in different AEZ with contrasting environmental conditions in Eastern Kenya. The aim of this study was to isolate, characterize morphologically and genetically and determine the diversity of the rhizobia populations associated with MAC 13 and MAC 64 climbing beans grown in different AEZ of Eastern Kenya.

## Materials and Methods

### Field Trapping of Rhizobia

Field trapping of rhizobia was carried out during the first rainy season between March and August 2015 in four different sites of Eastern Kenya with contrasting agro-climatic conditions using MAC 13 and MAC 64 climbing beans as the host plants. Eastern Kenya was chosen because of its high production potential of diverse common bean cultivars (Mburu et al., 2016). The four farmers’ fields used in rhizobia trapping were located in upper and lower midland AEZ of Embu (0.53°S, 37.45°E) and Tharaka Nithi (0.30°S, 38.06°E) Counties of Eastern Kenya. Three plots in each farm measuring 3 m × 3 m were prepared and climbing bean seeds were sown using the recommended spacing of 75 cm × 30 cm. Destructive sampling was done after 45 days since planting by randomly but carefully uprooting five plants within the mid-rows of each plot. A sterile clean spade was used to dig for approximately 15 cm sideways and up to a depth of about 20 cm. The clump of soil and the roots were uplifted carefully, placed in sterile aluminum foil where the nodules were detached from the roots and kept in screw-capped vials containing silica gel to prevent from desiccation. Extraction was done a day after nodules were harvested.

### Isolation of Rhizobia From the Root Nodules

Ten healthy and undamaged root nodules of MAC 13 and MAC 64 climbing beans from each site were selected and used in the isolation of rhizobia following the standard protocols described by Somasegaran and Hoben (1994). The nodules were washed, surface sterilized in 1% sodium hypochlorite solution for 3 min, rinsed in seven changes of sterile distilled water and then crushed with a sterilized glass rod (Muthini et al., 2014). A loop-full of the resulting suspension was streaked on Yeast extract mannitol agar (YEMA) supplemented with Congo red (0.00125 mg/kg) and incubated in the dark at 28°C for 3–5 days (Vincent, 1970). Emerging single colonies, which were typical of rhizobia species were sub-cultured by repeated streaking on YEMA, and YEMA containing bromothymol blue (BTB) plates (0.00125 mg/kg). A total of 41 rhizobia isolates were obtained. All isolates were maintained on screw-capped McCartney YEMA slant bottles and preserved at 4°C and on vials containing 25% glycerol-Yeast extract mannitol broth (YEMB) where they were preserved at -20°C.

### Morpho-Cultural and Biochemical Characterization of the Isolates

Using the standard microbiological techniques described by Somasegaran and Hoben (1994), all the isolates were characterized for selected morphological parameters such as colony size, shape, border, elevation, color, mucosity, transparency, and capacity to produce the exopolysaccharide gum (Liu, 2014; Muthini et al., 2014). Other tests that were carried out included Gram staining where young pure isolates (3–4 days old) cultured on YEMA were smeared on clean microscope slides. The wet smears were air-dried, heat fixed and then Gram stained as described by Beck et al. (1993). The prepared slides were observed under oil immersion on a compound light microscope. The production of acid or alkali was determined in YEMA medium containing bromothymol blue (YEMA-BTB). The plates were incubated at 28°C for 7 days in the dark. The isolates that changed the green color of YEMA-BTB to yellow were identified as acid producers and fast growers. Isolates that changed the YEMA-BTB medium to blue were considered as alkaline producers and slow growers. Lastly, rhizobia isolates were cultured in peptone glucose agar plates, incubated at 28°C for 4 days in the dark. The absence of bacteria growth was a clear indication of the presence of *Rhizobium*. Based on the differences in the observed morpho-cultural, Gram stain and growth features, the isolates were placed into different morphotype groups.

### Genotypic Characterization

The molecular study involved 41 rhizobia isolates and was carried out at the Department of Microbiology, Kenyatta University. The analysis of genetic relatedness of the native isolates from Eastern Kenya was carried out using amplified rDNA restriction analysis (ARDRA) of the 16s rRNA genes using three restriction enzymes *Hae* III, *EcoR* I and *Msp* I (Biolabs, England). Three reference strains; *Rhizobium tropici* CIAT 899, *Rhizobium etli* USDA 2667 strain and *Rhizobium leguminosarum* strain 446 were used. The three reference *Rhizobium* strains were obtained from the Microbiological Resource Centre (MIRCEN), University of Nairobi, Kenya.

### Genomic DNA Extraction of Rhizobia

The extraction of total genomic DNA of rhizobia isolates was carried out following modification of a method outlined by Young et al. (1991). The young rhizobia cultures grown in YEMA plates for 3 days in the dark at 28°C were re-suspended in sterile eppendorf tubes containing 400 μl of normal saline, vortexed for 20 s and centrifuged at 13,000 rpm for 10 min. The supernatant was poured out carefully leaving the cell pellets. The cell pellets were washed five times with normal saline to remove the slimy exopolysaccharide (EPS). The cell pellets were then harvested and re-suspended in 400 μl of cetyltrimethylammonium bromide (CTAB) lysis buffer, vortexed and incubated in a water bath at 65°C for 2 h (Silva et al., 2012). The tubes were intermittently inverted every 20 min during the incubation period. The DNA was extracted by adding equal volumes of phenol: chloroform: isoamyl alcohol solution (25:24:1), vortexed and centrifuged at 13,000 rpm for 10 min at 4°C (Berrada et al., 2012). The supernatant was transferred carefully to a new sterile 1.5 ml eppendorf tube where an equal volume (400 μl) of absolute ethanol was added and incubated at 4°C for 10 min to allow precipitation. The precipitated DNA was centrifuged at 13,000 rpm for 8 min and the supernatant was discarded. The DNA pellet was air-dried for 40 min and dissolved in 40 μl of DNase free water. It was then stored at -20°C.

### PCR Amplification of the 16S rRNA Genes

The PCR amplification was carried out in 30 μl reaction with 23.55 μl sterile PCR water, 3.0 μl buffer (Biolabs), 0.6 μl dNTP (10 mM), 0.3 μl of each Y1 and Y3 primers (10 μM), 0.6 μl 5% Tween 20, 0.15 μl Taq DNA polymerase (Biolabs) and 1.5 μl of DNA template. The sequences of the primers used for PCR amplification of the 16S rRNA gene were: Y1 forward primer (5′-TGGCTCAGAACGAACGCTGGCGGC-3′) that corresponds to positions 20–43 and Y3 reverse primer (5′-TACCTTGTTACGACTTCACCCCAGTC-3′) corresponding to positions 1482–1507 for 16S rDNA sequence of *Escherichia coli* (Young et al., 1991). A negative control without DNA was used. The DNA was amplified in a Techgene Thermal Cycler (Techne) programmed to run as follows; initial DNA denaturation at 94°C for 2 min; 35 cycles (denaturation at 94°C for 45 s, annealing at 62°C for 45 s and extension at 72°C for 2 min). Final extension was carried out at 72°C for 5 min. Amplified PCR products were visualized by gel electrophoresis on a 1% agarose gel. The stain used was SYBR-Green (Biolabs). DNA ladder of 1 Kb (Biolabs) was used to estimate the band sizes. The gel was run in Tris-Borate Ethylenediaminetetraacetic acid (0.5X TBE) buffer at 80 V for 50 min and was visualized using a UV trans-illuminator and photographed using a digital camera (Nikon).

### Amplified Ribosomal DNA Restriction Analysis of 16S rRNA Genes

Polymerase chain reaction products of the isolates and reference strains were digested using *Hae* III, *Eco*R I, and *Msp* I restriction enzymes (Biolabs) (Laguerre et al., 1996). A master-mix (10.0 μl) containing 3.8 μl PCR water, 1.0 μl reaction buffer, 0.2 μl restriction enzyme and 5.0 μl PCR amplicons were digested for 1 h at 37°C. The restriction digests (fragments) were then stained using SYBR-Green and separated on a 2% agarose gel. A 100 bp DNA ladder (Biolabs) was used to estimate the fragment sizes. The gel was run at 80 V for 50 min and was visualized using a UV trans-illuminator and photographed using a digital camera. The different band patterns were noted and the frequency of similar patterns was scored.

### Data Analysis

The data on rhizobia genetic diversity based on the different band patterns formed after PCR restriction were coded in binary form and analyzed as described by Silva et al. (2012) using Gene Alex Software version 6.5. Euclidean distance similarity, Nei’s unbiased genetic distance (Nei and Li, 1979), and single linkage (nearest neighbor) methods available in the PAST program (version 1.92) and Darwin software (version 6) were used to construct dendrograms for clustering the rhizobia isolates and to assess their diversity and similarity across the four AEZ of Eastern Kenya (Hammer et al., 2001). Shannon–Wiener diversity index (*H*) was also used to determine the rhizobia diversity in different AEZ of Eastern Kenya.

## Results

### Morpho-Cultural Characteristics of Isolates

**FIGURE 1:**
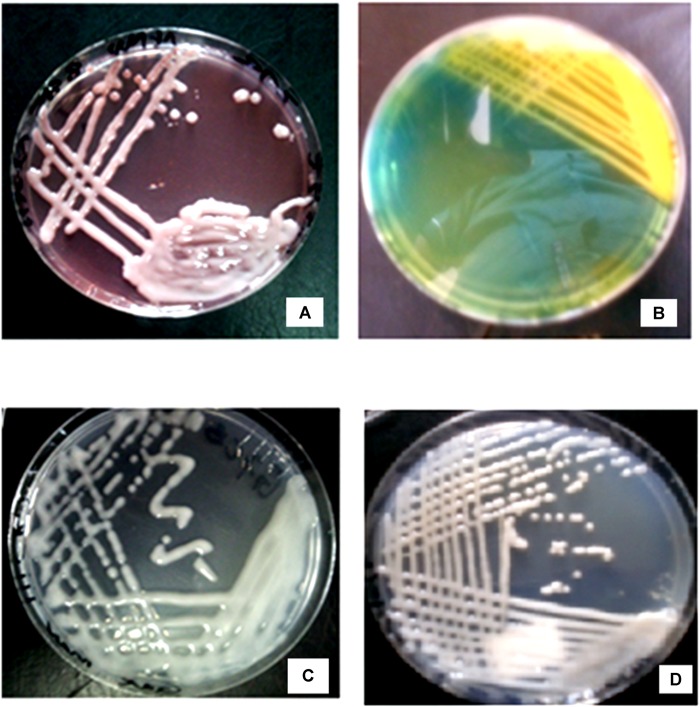
Morphological characteristics of rhizobia isolates obtained from field trapping. **(A)** Native rhizobia isolate EUM7 on YEMA with Congo red dye. **(B)** Acidic reaction of native rhizobia isolate ELM3 on YEMA with BTB. **(C)** Growth of native rhizobia isolate ELM5 on YEMA media. **(D)** Native rhizobia isolate TUM2 on YEMA media.

From the field trapping experiment using MAC 13 and MAC 64 climbing beans, 41 pure rhizobia isolates were obtained, which were placed in to nine distinct morphotype groups based on their morpho-cultural and biochemical features (**Table 1**). Morphotype III was the most abundant (29%) and morphotype II accounted for a partly 2% of the total number of isolates. All isolates were Gram negative rods and did not absorb Congo red dye when incubated in the dark on YEMA-CR medium (**Figure 1A**). Notably, all isolates turned BTB indicator from deep green to yellow when grown on YEMA-BTB indicating that the isolates were acid producers and fast growers (**Figure 1B**). The colony color varied with milky white, cream white, cream yellow and watery colonies being observed which were either opaque or translucent with either firm gummy or smooth mucoid texture (**Figure 1C**). Most of the isolates had a mucoid texture due to the production of excess EPSs. As expected, all isolates had an entire colony margin but the colony elevation varied consistently with convex and raised colonies being observed on YEMA media (**Figure 1D**). The morphological characteristics of the reference strains *Rhizobium tropici* CIAT 899 and *Rhizobium etli* USDA 2667 were closely similar to those of morphotype III while *Rhizobium leguminosarum* strain 446 closely resembled morphotype V (**Table 1**).

**Table 1:** Morpho-cultural and biochemical characteristics of the rhizobia isolates trapped from the study farms in Eastern Kenya.

Isolate characteristic	Morphotypes								
	
	I	II	III	IV	V	VI	VII	VIII	IX
CR absorption	Na	Na	Na	Na	Na	Na	Na	Na	Na
Peptone G growth	No	No	No	No	No	No	No	No	No
BTB reaction	Y	Y	Y	Y	Y	Y	Y	Y	Y
Gram reaction	-ve	-ve	-ve	-ve	-ve	-ve	-ve	-ve	-ve
Cell shape	Rod	Rod	Rod	Rod	Rod	Rod	Rod	Rod	Rod
Elevation	Cvx	Cvx	Cvx	Cvx	Cvx	Cvx	Cvx	Raised	Cvx
Margin	Entire	Entire	Entire	Entire	Entire	Entire	Entire	Entire	Entire
Colony color	Cy	Cw	Mw	Mw	Mw	Mw	W	Mw	Mw
Transparency	Op	Trl	Trl	Op	Op	Trl	Trl	Trl	Op
Colony texture	Fg	Fg	Sm	Sm	Fg	Sm	Sm	Fg	Sm
Percentage%	4	2	29	7	9	20	18	4	7


*Na, non-absorbing; Peptone G, Peptone Glucose growth; No, no growth; Y, yellow on BTB; -ve, negative; Cvx, convex elevation; Cy, Cream yellow; Cw, cream white; Mw, milky white; W, watery; Op, opaque; Trl, translucent; Fg, firm gummy; Sm, soft mucoid.*

### Genetic Diversity of Native Rhizobia Isolates From Eastern Kenya

#### DNA Extraction, PCR Amplification and ARDRA Analysis of 16S rRNA Genes

**FIGURE 2:**
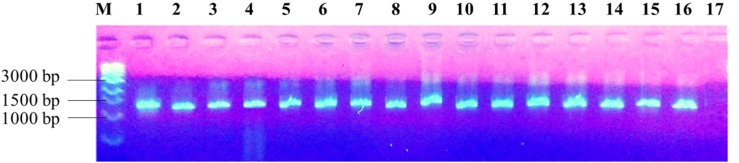
PCR amplified 16S rDNA of the isolates in 1% agarose gel. Lane M, 1 kb DNA ladder (Biolabs); Lanes 1–13, genomic DNA of selected native rhizobia isolates (ELM1, TUM2, ELM3, ELM4, ELM5, EUM6, EUM7, ELM8, ELM9, TLM10, TUM11, TLM12, and EUM13); Lane 14, CIAT 899; Lane 15, USDA 2667; Lane 16, strain 446; Lane 17, Negative control.

**FIGURE 3:**
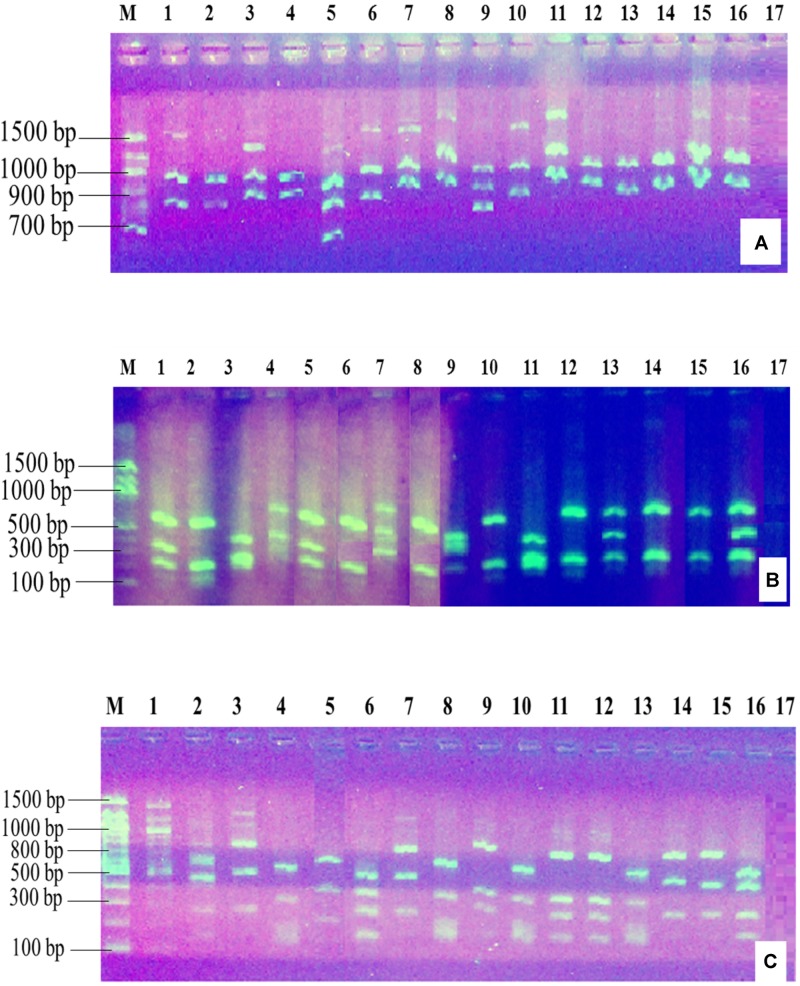
Gel electrophoresis of the restriction digestion products of 16S rDNA of selected native rhizobia isolates in 2% agarose gel. Lane M, 100 bp DNA ladder (Biolabs); Lanes 1–13, genomic DNA of native rhizobia isolates (ELM1, TUM2, ELM3, ELM4, ELM5, EUM6, EUM7, ELM8, ELM9, TLM10, TUM11, TLM12, and EUM13); Lane 14, CIAT 899; Lane 15, USDA 2667; Lane 16, strain 446; Lane 17, Negative control. **(A)** Restriction digestion with *EcoR* I; **(B)** restriction digestion with *Hae* III; **(C)** restriction digestion with *Msp* I.

The PCR amplification of the 16S rDNA region produced a single band of approximately 1500 bp (**Figure 2**). Restriction of the 16S rRNA amplicons of the climbing bean rhizobia isolates using enzymes *EcoR* I (**Figure 3A**), *Hae* III (**Figure 3B**) and *Msp* I (**Figure 3C**) produced multiple band patterns. Restriction enzyme *Msp* I produced the most diverse polymorphic patterns.

Based on ARDRA analysis of 16S rRNA amplicons, rhizobia population from ELM had the highest average number of different alleles (*Na* = 1.92 ± 0.08) and effective alleles (*Ne* = 1.55 ± 0.10) compared to other populations (**Table 2**). Rhizobia isolates from ELM had the highest percentage polymorphic loci (92.31% P) while isolates from EUM and TLM recorded the lowest at 61.54% P. The mean Shannon–Wiener diversity (*H*) estimate showed that the four rhizobia populations from Eastern Kenya were genetically diverse with rhizobia population from ELM having the highest genetic diversity estimate of *H* = 0.47 (**Table 2**). The rhizobia population from zone TLM recorded the lowest genetic diversity estimate of *H* = 0.31. The average expected heterozygosity (He) varied among the four populations and ranged from 0.21 for rhizobia population from TLM to 0.32 for ELM population (**Table 2**).

**Table 2:** Mean number of different alleles (Na), number of effective alleles (Ne), Shannon’s Information Index I (H), expected Heterozygosity (He) and percentage of Polymorphic Loci (% *P*) of native rhizobia populations from Eastern Kenya based on ARDRA analyses.

Population	Na	Ne	I(*H*)	He	% *P*
ELM	1.92 ± 0.08	1.55 ± 0.10	0.47 ± 0.07	0.32 ± 0.05	92.31
EUM	1.54 ± 0.18	1.39 ± 0.11	0.33 ± 0.08	0.22 ± 0.06	61.54
TLM	1.54 ± 0.18	1.37 ± 0.12	0.31 ± 0.08	0.21 ± 0.06	61.54
TUM	1.62 ± 0.18	1.41 ± 0.10	0.37 ± 0.08	0.25 ± 0.05	69.23


*ELM, Embu Lower Midland; EUM, Embu Upper Midland; TLM, Tharaka Nithi Lower Midland; TUM, Tharaka Nithi Upper Midland.*

Analysis of molecular variance (AMOVA) for the four rhizobia populations in Eastern Kenya showed a significantly high genetic variation (*P* = 0.0406; 97%) within populations (within a test agroecological zone). The variation among the rhizobia populations from the four AEZ (2.5%) and two Counties (0.5%) were, however, not significant (**Table 3**).

**Table 3:** Analysis of molecular variance (AMOVA) for 41 rhizobia isolates for the four populations from Eastern Kenya based on restriction digestion of 16S rDNA.

Source	Df	SS	MS	Est. Var.	% Mol Var.	*P*-value
Among regions	1	1.010	1.010	0.005	0.5	0.412
Among pops	2	1.812	0.906	0.000	2.5	0.870
Within pops	49	81.442	1.662	1.662	97.0	0.0406
Total	52	84.264		1.667	100	


*Df, degrees of freedom; SS, sum of squares; MS, mean square; Est. Var, estimated variance; % Mol. Var, percentage molecular variance; Pops, populations.*

**FIGURE 4:**
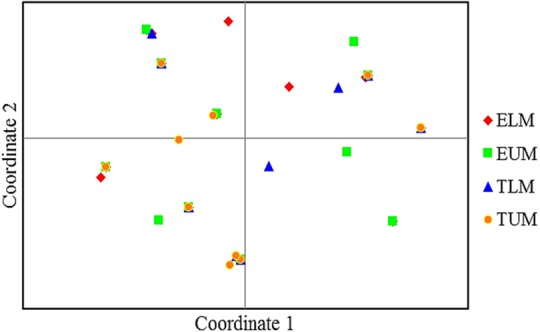
Principle coordinate analyses (PCA) of 41 native rhizobia isolates from Eastern Kenya based on ARDRA restriction patterns. Percentage variation explained by the first 2 coordinates; 1, 33.82%; 2, 20.74%. ELM, Embu Lower Midland; EUM, Embu Upper Midland; TLM, Tharaka Nithi Lower Midland; TUM, Tharaka Nithi Upper Midland.

The principal coordinate analysis (PCA) of 41 native isolates from the four zones of Eastern Kenya and three rhizobia reference strains showed considerable differentiation (**Figure 4**). Isolates from TUM zone were the most distributed and appeared in all the four quadrants, while isolates from ELM zone were the least distributed (**Figure 4**).

**FIGURE 5:**
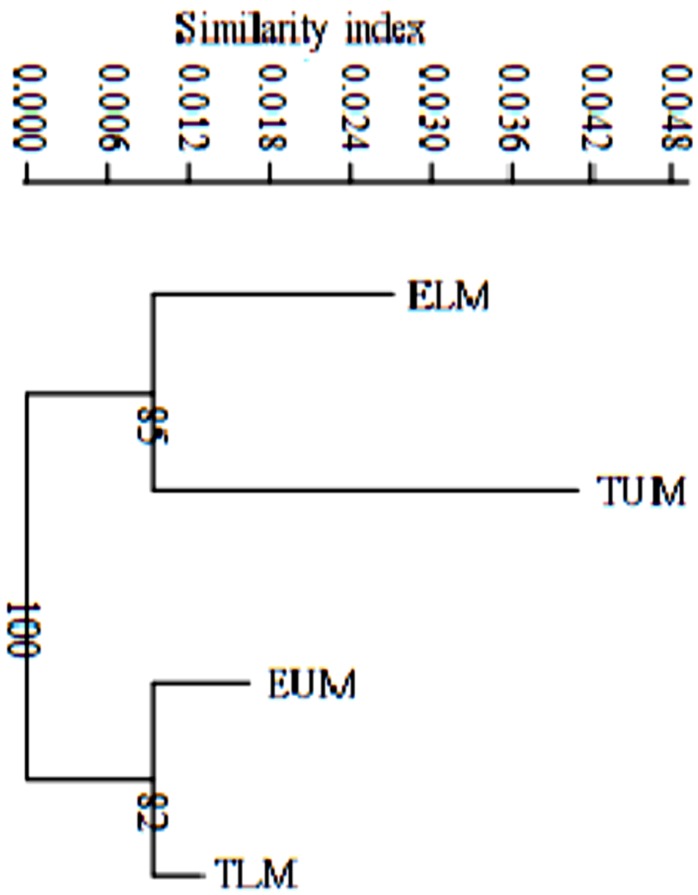
A neighbor joining dendrogram based on Nei’s unbiased genetic distance and Euclidian similarity index showing the genetic distance of four rhizobia populations from Eastern Kenya. Numbers shown at the nodes of the dendrogram indicate the percentage bootstrap support for 1000 iterations. ELM, Embu Lower Midland; EUM, Embu Upper Midland; TLM, Tharaka Nithi Lower Midland; TUM, Tharaka Nithi Upper Midland.

Based on the pairwise population matrix of Nei unbiased genetic distance and Euclidian similarity index, the neighbor joining dendrogram clustered rhizobia populations from Eastern Kenya into two main groups (**Figure 5**). Rhizobia populations from ELM and TUM clustered together with a bootstrap value of 85% while rhizobia populations from EUM and TLM clustered with a bootstrap value of 82%(**Figure 5**).

**FIGURE 6:**
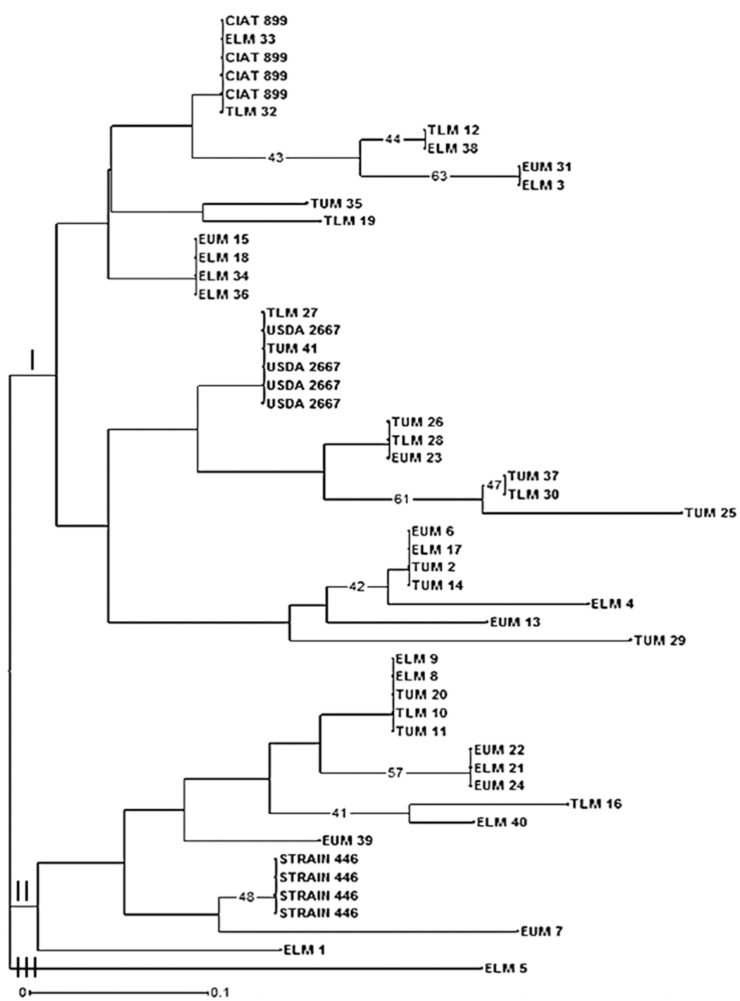
Phylogenetic relationship of 41 native rhizobia isolates from Eastern Kenya and three reference rhizobia strains (CIAT 899, USDA 2667, and strain 446) inferred using the Neighbor-Joining method. Numbers shown at the nodes of the dendrogram indicate the percentage bootstrap support for 1000 iterations. Only bootstrap values ≥ 40% are shown. Scale bar indicates number of substitutions per site. ELM, Embu Lower Midland; EUM, Embu Upper Midland; TLM, Tharaka Nithi Lower Midland; TUM, Tharaka Nithi Upper Midland.

Based on the genetic distance after amplified rDNA restriction analyses and Euclidian similarity index, the phylogenetic tree clustered the native rhizobia isolates into three main clusters I, II, and III (**Figure 6**). Cluster I comprised of majority of the isolates while cluster III had only one isolate ELM5. The reference strain *Rhizobium tropici* CIAT 899 which was placed in cluster I, clustered together with native rhizobia isolates ELM33 and TLM32, indicating their close genetic relationship. Native isolates TUM26, TLM28, and EUM23 clustered together despite originating from different AEZ of Eastern Kenya. *Rhizobium etli* USDA 2667 strain was also placed in cluster I and was grouped together with TLM27 and TUM 41 isolates (**Figure 6**). The native isolate EUM7 clustered closely to *Rhizobium leguminosarum* strain 446 in cluster II but in a separate sub-branch.

## Discussion

### Morphological Characteristics of Native Rhizobia Isolates

Gram staining results and growth on YEMA-CR and YEMA-BTB media, preliminary confirmed the standard morpho-cultural characteristics of *Rhizobium* species that nodulate with *P. vulgaris* L. as described by Vincent (1970) and Somasegaran and Hoben (1994). The Gram-negative rods observed and poor absorption of Congo red dye of the isolates on YEMA-CR further reinforced that the isolates were rhizobia (Beck et al., 1993). Their growth within 3–5 days and the color change on YEMA-BTB from deep green to yellow suggested that all isolates were fast growers and could probably fall under the genus *Rhizobium* (Al-mujahidy et al., 2013). The nine different morphotypes of rhizobia showed the diverse nature of the isolates colonizing nodules of MAC beans in Eastern Kenya. The results of this study on morphological and biochemical characterization of native rhizobia isolates nodulating common beans corresponded to the findings reported in other recent studies done in Kenya (Kawaka et al., 2014; Muthini et al., 2014) and in Ecuador (Torres-Gutiérrez et al., 2017).

Changes in temperatures, metal toxicity, pH and soil salinity are among the main factors restricting symbiotic nitrogen fixation in legume-rhizobia symbiosis and thus only those strains capable of tolerating the extreme conditions would survive (Berrada et al., 2012). The production of exopolysaccharides (EPS) by most of the isolates in this study could indicate their versatility to withstand physiological stress due to high temperatures, metal toxicity, low soil pH and salinity (Karthik et al., 2017). Soils in AEZ surrounding Mt. Kenya region are known to be slightly acidic due to excessive precipitation and soil erosion (Nyaga et al., 2014). Therefore, rhizobia strains native to Mt. Kenya and its environs are expected to have survival adaptations to counter the stressful soil and environmental conditions. Essentially, isolates exhibiting a wide adaptation to environmental stresses could be able to circumvent limiting factors and maintain a higher capacity for nitrogen fixation, thus, may be considered suitable candidates for rhizobia inoculum development (Kawaka et al., 2014; Torres-Gutiérrez et al., 2017).

### Genetic Diversity of Native Rhizobia Isolates From Eastern Kenya

The restriction digest using restriction enzymes *EcoR* I, *Hae* III, and *Msp* I showed highly polymorphic and distinct DNA fragment patterns indicating the divergence of the native rhizobia isolates in Eastern Kenya. The mean Shannon–Wiener diversity (*H*) estimate of rhizobia populations from Eastern Kenya based on genetic distance of ARDRA pattern analysis showed that the isolates were genetically diverse with rhizobia population from ELM zone having the highest genetic diversity estimate compared to TLM zone, which recorded the lowest diversity. This variation in rhizobia diversity could be due to the differences in agroclimatic and soil conditions of the four sites studied in Eastern Kenya. These results reinforce the promiscuous nature of *P. vulgaris* L. to nodulate with diverse strains of *Rhizobium* (Pohajda et al., 2016). Similarly, Wasike et al. (2009) reported a relatively higher diversity of indigenous bradyrhizobia in Western Kenya compared to that of Eastern Kenya as a result of agroecological differences between the two sites. Other factors such as cropping history, land use and host genotype have been attributed to the variation of rhizobia diversity in different parts of Central highlands of Kenya (Mwenda et al., 2011), which may also be the reason of the variation in our study.

The Pairwise Population Matrix of Nei unbiased genetic distance of rhizobia populations in Eastern Kenya showed a narrow range. These findings imply that the rhizobia populations, despite originating from different AEZ of Eastern Kenya, are closely related and may have a recent common ancestral origin (Mwenda et al., 2011). The narrow genetic distance could also possibly be as a result of the conserved nature of the 16S rRNA gene, which could not discriminate between closely related rhizobia species (Berrada et al., 2012). In addition, only three restriction enzymes were used in this study and thus could not produce highly distinct and polymorphic profiles that would produce a better diversity screening potential of the ARDRA technique (Silva et al., 2012). Thus, other molecular finger printing tools that have a higher resolving power, such as third generation gene sequencing and phylogenetic analysis, should be adopted. Dai et al. (2012) noted that the phylogenetic analyses of bacteria using 16S rDNA alone may not clearly show a distinctive relationship within and among the bacteria populations involved. In addition, horizontal gene transfer and genetic recombination could have possibly contributed to the limited genetic variation of rhizobia in Eastern Kenya. Similar findings have been reported by Ismail et al. (2013) who worked on *P. vulgaris* rhizobia populations and found a narrow genetic distance among isolates obtained from different sites in Egypt.

The analyses of molecular variance (AMOVA) based on amplified 16S rDNA restriction profiles showed a highly significant genetic variation of native rhizobia isolates within rhizobia populations and not among the four populations nor across the two regions (Counties) of Eastern Kenya. Based on ARDRA fingerprints, the low level of genetic differentiation of MAC bean rhizobia could suggest that the rhizobia population within the region is weakly structured. This could be due to the absence of physical barriers to limit gene flow (Muthini et al., 2014). Human activities such as transfer of plants, soils and the circulation of climbing bean seeds through trading within the region (Ramaekers et al., 2013) could have possibly contributed to the rhizobia genetic conservation in Eastern Kenya. Similar findings were reported by Elboutahiri et al. (2015) who observed a larger proportion of significant (*p* < 0.05) genetic variation distributed within regions (89%) than among regions (11%) in *S. meliloti* and *S. medicae* obtained from drought and salt affected regions of Morocco. Moreover, Rashid (2013) reported high variability of rhizobia isolates obtained from different geographical regions in Europe.

The principal coordinate analysis (PCA) also showed low level of genetic differentiation of native rhizobia isolates from Eastern Kenya and thus showing congruent results with the dendrogram. However, the grouping and distribution patterns of the native isolates did not correspond to the AEZ, and this is an indicative of the likelihood of a common evolutionary origin of the isolates. Similarly, Ismail et al. (2013), reported a PCA analysis of rhizobia isolates collected from different sites that did not correspond to the geographical locations in Egypt, and linked the genetic homogeneity of the isolates to the ribosomal gene recombination within and between rhizobia strains. A study by Wang et al. (2016) on biodiversity and biogeography of rhizobia associated with common beans in Shaanxi province, China, linked lateral gene transfer of symbiotic genes among different strains of nitrogen-fixing bacteria (*Agrobacterium*, *Bradyrhizobium*, *Rhizobium*, and *Ensifer*). Based on the evolutionary relationship of 41 isolates inferred using Neighbor-Joining method, some of the native isolates clustered closely with the three reference rhizobia strains used in this study. The phylogenetic tree showed a diverse genetic variation among the native rhizobia isolates as evident by the different clusters and sub-clusters.

From the phylogenetic tree, most of the native rhizobia isolates clustered together with *Rhizobium tropici* CIAT 899 and *R. etli* USDA 2667 in cluster I. For instance, native isolates TLM 27 and TUM 41 clustered together with *R. etli* USDA 2667 while isolate TLM 32 clustered with *R. tropici* CIAT 899. These indicate the close genetic relationship between some of the native rhizobia isolates to *R. tropici* CIAT 899, *R. etli* USDA 2667 and *R. leguminosarum* strain 446 (Wang et al., 2016). Other studies in Kenya have shown the dominance of these *Rhizobium* strains in Kenyan soil (Anyango et al., 1995; Mwenda et al., 2011; Onyango et al., 2015). Based on ARDRA analysis, native isolates TUM 26, TLM 28, and EUM 23 clustered together despite originating from different AEZ of Eastern Kenya. This indicates the close genetic distance between some of the rhizobia isolates native to different AEZ of Eastern Kenya. There is also a possibility of such isolates having originated from the same genetic background. However, this may not be conclusive unless other molecular tools used in studying genetic diversity such as sequencing and phylogenetic analyses are employed.

## Conclusion

In this study, 41 rhizobia isolates were obtained from the root nodules of mid-altitude climbing (MAC) beans and based on morpho-cultural and biochemical characteristics, the isolates grouped into nine morphotypes indicating the diverse nature of rhizobia nodulating with MAC beans. Cluster analysis based on genetic characteristics obtained after PCR-ARDRA profiling, showed a larger proportion of significant (*p* < 0.05) genetic variation distributed within populations (97%) than among populations (2.5%) in four AEZ of Eastern Kenya. Despite the high diversity shown within the rhizobia population, the Pairwise Population Matrix of Nei unbiased genetic distance of rhizobia populations in the study region showed a narrow range, which is an indication of a weakly structured genetic population and a highly conserved genetic structure of rhizobia within the study area. Further molecular studies using either full or partial gene sequences of bacterial genome, which is more sensitive and has a higher resolution power, needs to be carried out to establish the true diversity of native rhizobia isolates up to the species and strain levels.

## Author Contributions

JK, OO, JM, and EN conceived and designed the research and data collection tools and participated in drafting the manuscript. GK and SM collected the data, participated in data analyses, and wrote the manuscript. EN performed the data analyses. All authors read and approved the final manuscript.

## Conflict of Interest Statement

The authors declare that the research was conducted in the absence of any commercial or financial relationships that could be construed as a potential conflict of interest.

## Abbreviations

AEZ: agroecological zones
ELM: Embu Lower Midland
EUM: Embu Upper Midland
MAC: Mid-altitude climbers
TLM: Tharaka Nithi Lower Midland
TUM: Tharaka Nithi Upper Midland
